# Does Adjuvant Treatment with Chinese Herbal Medicine to Antidiabetic Agents Have Additional Benefits in Patients with Type 2 Diabetes? A System Review and Meta-Analysis of Randomized Controlled Trials

**DOI:** 10.1155/2019/1825750

**Published:** 2019-05-06

**Authors:** De Jin, Jiaxing Tian, Qi Bao, Haiyu Zhang, Qiyou Ding, Fengmei Lian, Tong Xiao-lin

**Affiliations:** Guang An Men Hospital, China Academy of Chinese Medical Sciences, Beijing, China

## Abstract

**Introduction:**

In the present meta-analysis, we aimed to determine the effects of adjuvant treatment with Chinese herbal medicine (CHM) on antidiabetic agents having additional benefits in patients with type 2 diabetes.

**Methods:**

Randomized controlled trials were identified by searching the Cochrane Library, PUBMED, EMBASE, MEDLINE, the China National Knowledge Internet, Web of Science, Global Health, International Pharmaceutical Abstracts and the China biology medicine, Wanfang, and VIP databases. The intervention group received CHM as add-on treatment to antidiabetic agents therapy, and the control group received placebos in addition to antidiabetic agents or antidiabetic agents alone. We assessed pooled data, including weighted mean differences and 95% confidence intervals (CIs) using a random-effects model.

**Results:**

A total of 125 randomized controlled trials were included. 10 articles were included based on literature screening. All trials contrasted Chinese herbal medicines or Chinese herbal medicines + antidiabetic agents with placebo or antidiabetic agents + placebo and included a total of 2004 individuals with T2DM. All selected trials displayed evidence of high methodological quality and possessed a low risk of bias. Meta-analysis of the trials demonstrated that Chinese herbal medicines resulted in a more favorable blood glucose profile in contrast to placebo (P<0.05). The total efficacy rate differed significantly between the two groups (P<0.001). All ten included studies reported the occurrence of tolerable adverse effects.

**Conclusions:**

The results showed that in the intervention group, greater reductions were achieved for glucose control and body weight. The combined use of drugs improves the curative effect and has fewer adverse events and has additional benefits in patients with type 2 diabetes. This trial is registered with PROSPERO (CRD42018093867).

## 1. Introduction

Type 2 diabetes mellitus (T2DM) is a noncommunicable disease that has seen a sharp rise in its global prevalence. The latest International Diabetes Federation (IDF) survey revealed that there were approximately 425 million patients with T2DM worldwide in 2017, with this number predicted to grossly surpass the previous estimation of more than 645 million patients by 2045. With 109.6 million patients, China is home to the largest numbers of diabetics* (IDF Diabetes Atlas.8th ed.)*. An epidemiological survey from China found that the estimated overall prevalence of diabetes was 10.9% amongst adults living in the country [1]. It is well known that diabetic individuals possess higher risks of comorbid illnesses, higher functional disability, and rates of premature death, compared to subjects without the condition. Patients with T2DM also suffer from a myriad of complications [2]. Several clinical trials have shown that good glycemic control is essential for maintaining the health of patients with T2DM [3, 4]. Guidelines issued by the American Diabetes Association, European Association, and Chinese Diabetes Society for the Study of Diabetes recommend lifestyle interventions and oral glucose-lowering drugs (beginning with metformin) for managing HbA1c levels in patients with T2DM [5]. Additional antidiabetic medications such as sulfonylureas, thiazolidinediones, and insulin are implemented into treatment regimens when metformin monotherapy fails to achieve glycemic targets within three months [6]. Nevertheless, several of these medications possess side effects such as weight gain or hypoglycaemia that may increase insulin resistance [7].

Chinese herbal medicines (CHMs) have a long history of usage by Chinese medicinal practitioners in China and have been subjected to empirical investigations to determine its utility for treating T2DM. Several clinical trials [8–11] demonstrate the ability of CHMs to provide consistent glycemic control along with added benefits such as decreasing blood glucose levels, ameliorating insulin resistance and boosting pancreatic islets function, promoting weight loss, and having a low incidence of adverse events. Pharmacological studies demonstrated that CHMs may have the potential to partially restore islet beta cell function lost due to trauma [12–14], increase insulin secretion [15, 16], and strengthen peripheral glucose uptake [17, 18].

Given the significant medical and socioeconomic burden of T2DM, several controlled studies were performed to explore the safety and efficacy of CHMs for T2DM. A 2004 Cochrane Review [19] evaluated the efficacy of CHMs for T2DM and reported that certain compounds may be able to lower blood glucose levels in T2DM. However, these findings should be interpreted with caution due to the small number of trials with low methodological quality. High-quality trials are warranted to properly investigate the utility of CHMs in T2DM, and, indeed, several have been performed since then. This systematic review and meta-analysis aims to evaluate the safety and efficacy of CHMs for treating patients with T2DM. With this data, we are able to better conclude if CHMs is able to function as a complementary therapy for T2DM and to clarify whether CHMs in combination with antidiabetic agents or CHMs treated alone are able to confer a hypoglycemic effect in T2DM.

## 2. Methods

The Preferred Reporting Items for Systematic Reviews and Meta Analyses (PRISMA) guidelines were used to guide the execution and reporting of this review. Systematic review registration no. is PROSPERO (CRD42018093867). This work does not need ethics approval due to requirement of PRISMA (Supplementary File 1).

### 2.1. Database and Search Strategies

Relevant reports were extracted from the following databases: MEDLINE, EMBASE, PUBMED, the Cochrane Library, China National Knowledge Internet, Global Health, International Pharmaceutical Abstracts, Web of Science and the China biology medicine, Wanfang, and VIP databases. The literature search strategy (Supplementary File 2) was constructed based on the following facets: the study design (randomized clinical trial), the intervention (Chinese herbal medicine), and the condition (T2DM). The search terms utilized were (herbal medicine OR herbs OR Chinese herbal medicine OR Chinese medicinal herb OR Chinese patent medicine) and (type 2 diabetes mellitus OR type 2 diabetes OR 2 diabetes OR DM) and (randomized clinical trial OR randomized OR RCT). Journals published either in Chinese or English were included in the final results.

### 2.2. Inclusion Criteria

#### 2.2.1. Types of Studies

All studies selected were designed to be clinical randomized controlled trials (RCTs).

#### 2.2.2. Types of Participants

Participants were of any ethnic origin, gender, and age and the definition of T2DM used in the studies was required to be in accordance with either the World Health Organization criteria [20] or the American Diabetes Association criteria [6].

#### 2.2.3. Types of Interventions

Participants in all trials either received CHMs treatment or a control medication. Patients in the CHMs treatment received CHMs + antidiabetic agents while those in the control group received either a placebo + antidiabetic agents or antidiabetic agents in isolation. Medication dosages of drugs across both intervention and control groups were required to be similar.

#### 2.2.4. Types of Outcome Measures

The primary measures of outcomes of interest were HbA1c (glycosylated hemoglobin) after treatment. Secondary outcome measurements were 2-hour postprandial blood glucose (2hPG), fasting blood glucose (FBG), and Body Mass Index (BMI). Medication safety data, if reported by the trial, was defined as the frequency of adverse events that occurred during the trial as a result of the medication.

### 2.3. Data Extraction and Management

Three independent authors (Jin De, Jiaxing Tian, and Qi Bao) screened the titles, abstracts, and contexts of each study for inclusion/exclusion with identical selection criterion. Incongruent opinions were first discussed, with a third party (Fengmei Lian) consulted for further resolution of any disagreements. The following data were extracted: trial characteristics (title, authors, year); baseline population characteristics (sample size, gender, age); interventions (treatment and control choice, dosage, and regimen); and outcomes (outcome measures, duration of patient follow-up, adverse events)

### 2.4. Statistical Analysis

The RevMan5.2 software provided by the Cochrane Collaboration was used to pool all reported outcomes [21]. Categorical outcomes were analyzed to determine relative risks (RRs) and accompanying 95% Cis; continuous outcomes were analyzed to determine WMDs (weighted mean differences) and accompanying 95% confidence intervals (CIs). Study heterogeneity was assessed with the I2 statistical method. If the I2 value was <50%, the fixed-effects (FE) model was adopted; values of <50% were subjected to the random-effect model. Corresponding authors were contacted directly for clarification should there be incomplete or missing primary outcome data (e.g., standard deviation (SD) and variance measures). When necessary, SD values were derived from SE or CI based on methods outlined in the Cochrane Handbook. Potential publication bias was assessed via a funnel plot.

### 2.5. Study Selection

**Figure 1:**
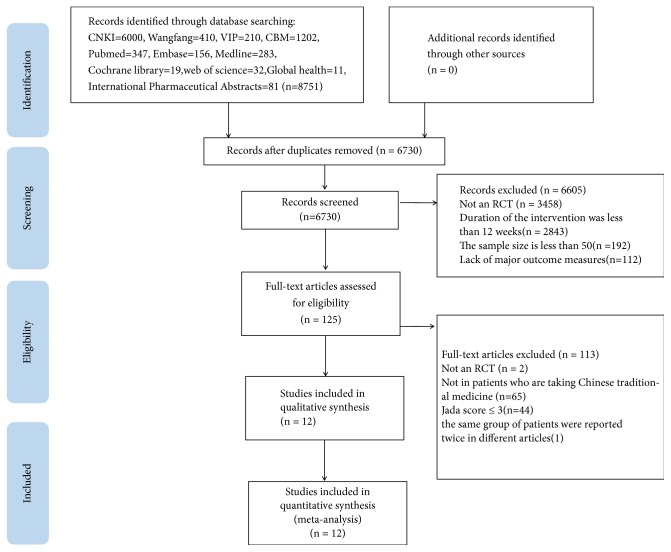
The screening process summarized in a flow diagram.

A total of 6730 potentially relevant articles were identified. A total of 6605 articles were excluded on the basis of study design, a short duration of intervention of less than 3 months, a lack of major outcome measures or a lack of a comparison group. Out of these 6730 articles, we evaluated 125 full-text articles for inclusion. 115 articles were excluded as they were not designed as RCT, not utilizing CHMs or were duplicates. A final count of 10 RCTs comprising 2004 participants met our inclusion criteria [8, 10, 11, 22–28].  Figure 1 depicts the screening process in the form of a flow diagram (Figure 1).

### 2.6. Characteristics of Included Studies

2004 participants between the age of 18 and 62 years old were included in all eligible RCTs.  Table 1 presents a summary of the characteristics of the studies, including author and publication year, sample size, participant age, course of disease, intervention and dosage, treatment duration, and outcome measures. All ten trials contrasted two groups of patients, one of which was designated as the “treatment group” (abbreviated to“T”in Table 1; these subjects were treated with CHMs + antidiabetic agents) and the other being a“control group”(abbreviated to “C”in Table 1; these subjects were treated with antidiabetic agents + placebo or antidiabetic agents only).

### 2.7. Methodological Quality Assessment and Evidence Quality

**Figure 2:**
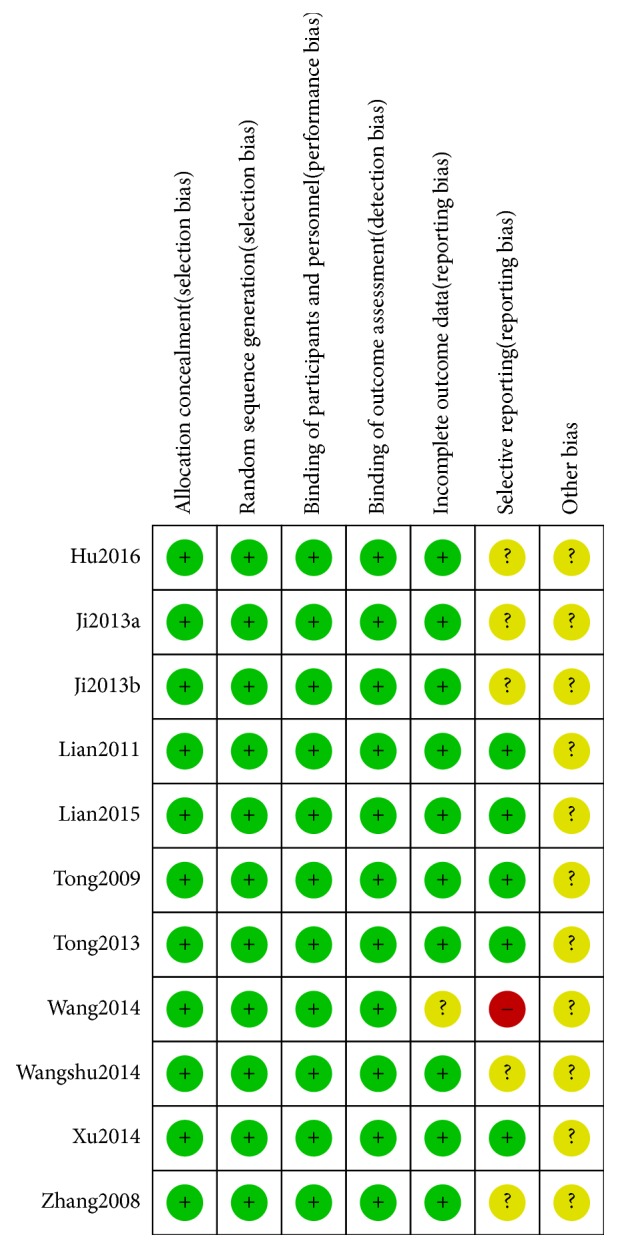
Quality assessment of the included trials-risk of bias graph.

**Figure 3:**
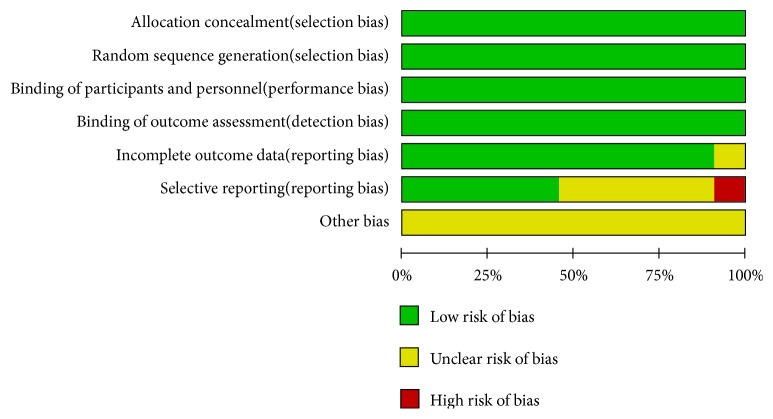
Quality assessment of the included trials-risk of bias summary.

Two independent reviewers (Hai-yu Zhang and Qiyou Ding) reviewed the quality of each included trial based on the Cochrane Collaboration's tool to assess the risk of bias. Randomized controlled trials were assessed using the Cochran Risk of Bias tool [29] which evaluates random sequence generation, allocation concealment, blinding, incomplete data regarding outcome, selective reporting, and other bias. All criteria on the list were ranked on a scale of low, unclear, or high bias risk. Any disputes between analyses were discussed until resolution was achieved. Figures 2 and 3 depict a detailed overview of how each study scored in each category of bias. Grading of Recommendations Assessment, Development and Evaluation (GRADE) [30] was used to assess the quality of the evidence for each outcome. According to GRADE, RCTs are considered higher-quality evidence, and observational studies are of lower quality. The risk of bias (in individual studies), inconsistency (heterogeneity in estimates of effect across studies), indirectness (related to the question or due to intransitivity), imprecision, and publication bias were addressed (Figure 8).

### 2.8. Description of the CHMs

Table 2 depicts a summary of all types of CHMs utilized across the included studies. 50 different types of CHMs were described. The top 16 most frequently utilized herbs that were used more than three times across the studies were* goldthread root (Coptis chinensis), Rehmannia glutinosa (Rehmanniae Radix Praeparata), Panax ginseng (Panax Ginseng C.A.Mey.), Lobed Kudzuvine Root (Radix Puerariae), Milkvetch Root (Hedysarum Multijugum Maxim.), Trichosanthes kirilowii Maxim(Trichosanthis Radix), Bupleurum chinense (Radix Bupleuri), Rhubarb (Radix Rhei Et Rhizome), Lycium chinense Mill root (Lycii Cortex), Wolfiporia cocos (Poria Cocos(Schw.) Wolf.), Scutellaria baicalensis (Scutellaria baicalensis Georgi), nagaimo (Dioscorea opposita), Cornus officinalis Sieb. et Zucc (Cornus officinalis), Atractylodes Lancea (Thunb.) DC. (Rhizoma Atractylodis), Prunus mume (Dark Plum Fruit), and Rhizoma Anemarrhenae (Anemarrhena asphodeloides)* (Table 3).

### 2.9. Meta-Analysis

#### 2.9.1. Change in HbA1c

**Figure 4:**
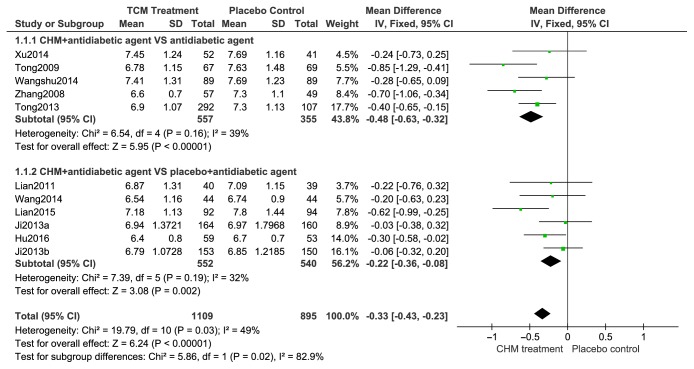
Forest plots of comparison of HAb1c for CHM plus antidiabetic agents therapy versus antidiabetic agents alone.

Figure 4 depicts the change in HbA1c values. All 10 trials (n=2004) reported treatment groups to have significantly changed HbA1c in contrast to the control groups. All HbA1c levels were relatively homogenous across trials, allowing for a fixed-effects model to be employed for statistical analysis. Change in HbA1c caused by CHMs was compared to the corresponding HbA1c values in the control group via subgroup analysis. The impact of CHMs on HbA1c levels were significantly different (n=912; MD, -0.48; 95% CI, -0.63 to -0.32; p<0.00001; I2=39%) compared to patients using placebos alone in 5 trials. The remaining five trials (indicated as “Add on”) compared change in HbA1c conferred by use of either CHMs + antidiabetic drugs or placebo + antidiabetic drugs (n=1092). These trials also demonstrated that a combination of CHMs + antidiabetic drugs was able to result in significantly reduced HbA1c levels (MD, -0.22; 95% CI, -0.36 to -0.08, p <0.01; I2=32%) in contrast to placebo.

#### 2.9.2. FBG

**Figure 5:**
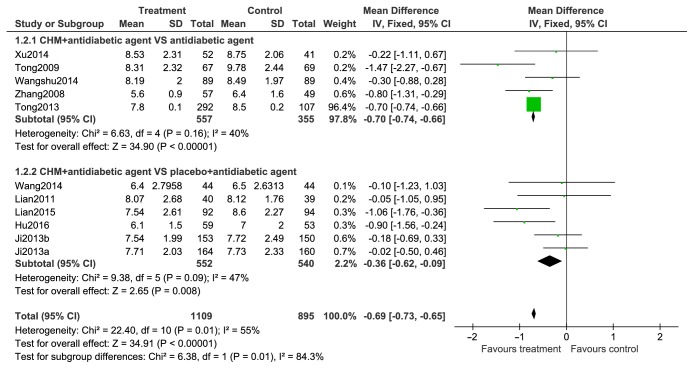
Forest plots of comparison of FBG for CHM plus antidiabetic agents therapy versus antidiabetic agents alone.

FBG levels are contrasted in Figure 5. All 10 trials (n=2004) included FBG levels as an outcome. Given that the results between trials were significantly heterogenous, a random-effects model was used to analyze trials. Subgroup analyses were performed to contrast FBG level differences between the treatment and control groups. In five trials, CHMs were found to significantly attenuate FBG levels in comparison to the control group (n=912; MD, –0.69; 95% CI, –0.96 to –0.43; p<0.00001; I2=40%). Subsequent analysis of the remaining five trials found that a combination of CHMs + antidiabetic drugs yielded significant differences in FBG levels when compared to patients taking a combination of placebo + antidiabetic drugs (n =1092; MD, –0.39; 95% CI, –0.78 to -0.01; p=0.04; I2=47%).

#### 2.9.3. 2hPG

**Figure 6:**
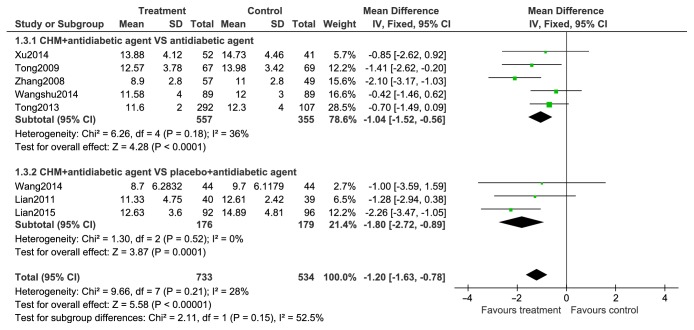
Forest plots of comparison of 2hPG for CHM plus antidiabetic agents therapy versus antidiabetic agents alone.

2-hour PG levels are compared in Figure 6. 2hPG levels were evaluated in 8 trials. A random-effects model was utilized for statistical analysis as there was significant result heterogeneity across trials. The impact of treatment and control groups of 2hPG levels were further assessed via subgroup analyses. 2hPG levels were compared between CHMs and placebo alone in eight trials, with results favoring treatment with CHMs (n=912; MD, –1.07; 95% CI, –1.69 to –0.46; p=0.0007; I2= 36%). 2hPG levels were also significantly different between groups using CHMs + antidiabetic drugs and groups using placebo + antidiabetic drugs (n=355; MD, –1.80; 95% CI, –2.72 to –0.89; p=0.0001; I2=0%).

#### 2.9.4. BMI

**Figure 7:**
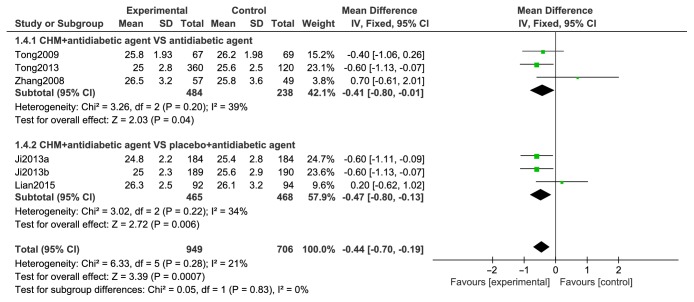
Forest plots of comparison of BMI for CHM plus antidiabetic agents therapy versus antidiabetic agents alone.

BMI data from six studies appear in Figure 7 and comparisons of BMI with CHMs and antidiabetic agents with controls showed heterogeneity between trials (I2=0%) and a statistical difference between two groups for BMI variations (n=1654, MD=-0.04; 95% CI-0.70 to -0.19) (Figure 7).

### 2.10. Adverse Events

Adverse events were reported in four trials, with two of these reporting the absence of adverse events (Table 4). Statistical analysis of the two trials that reported adverse events found no significant differences between the rate of adverse events between the treatment and placebo groups (n=1224, RR:0.70; 95% CI 0.37-1.29). Routine blood, renal, liver, and urine analysis as well as ECG findings demonstrated no significant difference between pre- and posttreatment values.

### 2.11. Assessment of Quality of Evidence

Table 5 showed overall evidence quality for each outcome (except adverse events) using the GRADE method. Generally, evidence quality was high for CHMs and diabetes, and FBG, 2hPG, and FBG

### 2.12. Publication Bias

**Figure 8:**
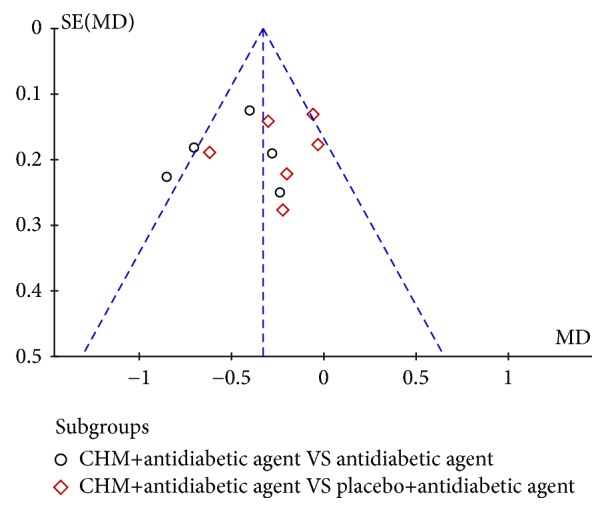
Funnel plot of the trials that compared treatment group with control group.

A funnel plot was used to express the publication bias. There were 10 trials included in the funnel plot, and no significant asymmetry was observed (Figure 8).

## 3. Discussion

### 3.1. Summary of the Evidence and Explanation of the Results

Development of antidiabetic medication has expanded tremendously in the recent decades. The most common and most frequently studied oral antidiabetic agents are metformin, sulfonylureas, and thiazolidinediones. The Chinese Diabetes Society (CDS), the American Diabetes Association (ADA), and the European Diabetes Association for the Study of Diabetes (EASD) all unanimously recommend the use of such agents for the initial treatment of T2DM [31]. The role of CHMs in primary healthcare in China and other Asian countries has been increasingly prominent [32–35], especially in the management of T2DM. A previous meta-analysis in 2002 Cochrane included 66 randomized controlled (8803 participants) studying the use of CHMs in type 2 diabetes; some herbal medicines show hypoglycaemic effects in type 2 diabetes. However, these articles with low methodological quality, small sample size, and lack of major evaluation outcomes (HbA1c) may add some difficulty in precisely evaluating the efficacy and safety of CHMs. We performed a meta-analysis RCT (High Quality) to evaluate the efficacy and safety of CHMs plus antidiabetic agents in T2DM. Compared with the placebo + antidiabetic agents or antidiabetic agents alone, the CHMs plus antidiabetic agents typically reduced HbA1c, 2hPG, and FPG levels in T2DM and resulted in a higher proportion of subjects that reduced weight.

Obesity or overweight is a risk factor for a wide range of chronic diseases, including coronary heart disease, diabetes, stroke, and cancer which associated with an increased risk of all causes of mortality. Weight control is necessary for patient with T2DM. Many antidiabetic drugs, such as thiazolidinediones, glinides, and sulphonylureas might lead to weight gain; it is vital for the patient with T2DM to select the appropriate drug. CHMs plus antidiabetic agents treatment resulted in an obvious reduction in BMI compared with the placebo + antidiabetic agents or antidiabetic agents monotherapy and might expand application range for antidiabetic drugs which might lead to weight gain.

In diabetes management, hypoglycemia is the most common adverse challenge. Interestingly, compared with the control group, the event of hypoglycemia was significantly lower in CHMs group than that in the placebo + antidiabetic agents or antidiabetic agents in subjects. The combination therapy was also associated with fewer adverse events (AEs). Our results showed the superiority of CHMs plus antidiabetic agents in patients with T2DM. CHMs are possibility beneficial when used as drug-combination therapy for patients with T2DM. However, the included antidiabetic drugs were only sulphonylureas, metformin, glinides, and thiazolidinediones. At present, there is lack of evidence associated with drug combination of other glucose-lowering drugs, such as GLP-1, DPP-4, and SGLT2. More RCTs are needed to evaluate the efficacy and safety of CHMs plus newly developed hypoglycemic agents.

## 4. Limitations

The current systematic review and meta-analysis possesses a number of limitations. Firstly, trial registration with the WHO International Clinical Trials Registry Platform was not obtained for half of all included studies. In September 2007, the International Committee of Medical Journal Editors (ICMJE) began implementing a mandatory requirement that all clinical trials are to be registered [36]. Therefore, all trial protocols could not be confirmed to be free from biased reporting. Secondly, there was significant clinical heterogeneity across the included studies, with large variations in CHMs formulation, dosage, and treatment. Furthermore, there was insufficient detail available in the studies to adequately confirm manufacturing quality and therefore raised concerns regarding CHMs formulation consistency. Future studies would benefit from including descriptions of quality control, medication formulation, administration, and dosage [37].

Thirdly, several studies lacked formal estimation of sample sizes, leading to inadequate cohort sizes. This reduces the reliability and accuracy of obtained data and raises the risk of potentially overestimating the benefits of intervention [38, 39]. Based on these reasons, results obtained from this meta-analysis should be interpreted with caution.

### 4.1. Implications for Practice

Based on the relative clinical heterogeneity of the included studies [I2=49%, Test for overall effect: Z=6.24(P<0.00001)] in reporting the effects of CHMs on HbA1c levels, along with the tolerability of CHMs across different patient cohorts, it could be concluded that CHMs may have the potential to serve as a safe, alternative, and complementary treatment for T2DM patients.

### 4.2. Implications for Research

T2DM is a condition that is being increasingly managed with CHMs, warranting larger clinical trials in this field. The most frequently used herbs such as* goldthread root (Coptis chinensis), Rehmannia glutinosa (Rehmanniae Radix Praeparata), Panax ginseng (Panax Ginseng C.A.Mey.), Lobed Kudzuvine Root (Radix Puerariae), Milkvetch Root (Hedysarum Multijugum Maxim.), Trichosanthes kirilowii Maxim (Trichosanthis Radix), Bupleurum chinense (Radix Bupleuri), Rhubarb (Radix Rhei Et Rhizome), Lycium chinense Mill root (Lycii Cortex), Wolfiporia cocos (Poria Cocos (Schw.) Wolf.), Scutellaria baicalensis (Scutellaria baicalensis Georgi), nagaimo (Dioscorea opposita), Cornus officinalis Sieb. et Zucc (Cornus officinalis), Atractylodes Lancea (Thunb.) DC. (Rhizoma Atractylodis), Prunus mume (Dark Plum Fruit), Rhizoma Anemarrhenae (Anemarrhena asphodeloides)* may contribute in the formation of a basic prescription for treating T2DM. Further trials should consider designing protocols in accordance with the CONSORT 2017 statement [40], a 25-item checklist assessing trial quality. Lastly, detailed information regarding CHMs preparations, quality of manufacturing, route of administration and dosage should be included for reference in future studies [37].

## 5. Conclusion

The current systematic review and meta-analysis has highlighted the benefits and safety of CHMs when used by patients with T2DM. CHMs treatment conferred clinically and statistically significant decreases in HbA1c, FPG, 2hPG, and BMI levels in T2DM patients. Therefore, certain CHMs are possibly beneficial when used as drug-combination therapy for patients with T2DM. However, our findings should be interpreted with caution because of the limitations of the study. More rigorous RCTs are essential in allowing closer assessment of the potential benefits and safety profile of CHMs in the management of patients with T2DM.

## Figures and Tables

**Table 1:** Characteristics of included studies.

Study ID	Subjects (T/C)	Age (years) (T/C)	Diagnostic criteria	Intervention		Main outcome measures	Course of Treatment	Language of Publication
				T	C			
Zhang 2008	57/49	T:51±9 C: 51±10	1999 WHO	berberine+ Metformin	placebo+ Metformin	HbA1c FBG 2hPG	12 weeks	English

Tong 2013	292/107	T:54.4±7.7 C:54.5±7.6	1999 WHO	TM81+ Metformin	placebo+ Metformin	HbA1c FBG 2hPG	12 weeks	English

Ji2013a	164/160	T:54±9 C:54±8	1999 WHO	Xiaoke pills+ Glibenclamide	Glibenclamide	HbA1c FBG, 2hPG	48 weeks	English

Ji2013b	153/150	T:54±8 C:55±9	1999 WHO	Xiaoke pills+ Glibenclamide+ Metformin	Glibenclamide + Metformin	HbA1c FBG, 2hPG	48 weeks	English

Lian 2015	92/94	T:55.18±9.13 C:55.81±9.93	1999 WHO	Jinlida + Metformin	Placebo + Metformin	HbA1c FBG, 2hPG	12 weeks	English

Hu 2016	59/53	T:56.3±11.1 C:54.9±10.3	1999 WHO	Jian yu tang Kang + Metformin	Placebo + Metformin	HbA1c FBG, 2hPG	26 weeks	English

Xu 2014	52/41	T:55.06±7.49 C: 50.7±9.40	1999 WHO	Ge Gen Qin Lian+ Metformin	placebo+ Metformin	HbA1c FBG, 2hPG	12 weeks	English

Wangsh 2014	89/89	T:56.27±9.8 C:55. 78±9.17	1999 WHO	Tang ke+ Metformin	Placebo+ Metformin	HbA1c FBG, 2hPG	12 weeks	Chinese

Tong 2009	67/69	T:52.93±6.58 C:52.4±8.61	1999 WHO	TangMinling pills+ Metformin	Placebo+ Metformin	HbA1c FBG, 2hPG	12 weeks	Chinese

Lian 2011	40/39	T:53.3±6.98 C:56.4±9.00	1999 WHO	Tian Qi Jiang Tang + Metformin	Placebo + Metformin	HbA1c FBG, 2hPG	12 weeks	Chinese

Wang 2014	49/44	T:59.00±6.5 C:63.00±5.6	1999 WHO	Liuwei Dihuang Combined With Ginkgo Leaf+ Metformin	placebo+ Metformin	HbA1c FBG, 2hPG	96 weeks	Chinese

**Table 2:** Analysis of the top 16 frequently used Chinese herb medicines in treatment of T_2_DM.

Herb name English (Latin)	Frequency	frequency %	Cumulative percentiles %
goldthread root (Coptis chinensis)	7	8.64	8.64
Rehmannia glutinosa (Rehmanniae Radix Praeparata)	5	6.17	14.81
Panax ginseng (Panax Ginseng C. A. Mey.)	5	6.17	20.98
Lobed Kudzuvine Root (Radix Puerariae)	3	3.70	24.68
Milkvetch Root (Hedysarum Multijugum Maxim.)	3	3.70	28.38
Trichosanthes kirilowii Maxim (Trichosanthis Radix)	3	3.70	32.08
Bupleurum chinense (Radix Bupleuri)	2	2.47	34.55
Rhubarb (Radix Rhei Et Rhizome)	2	2.47	37.02
Lycium chinense Mill root (Lycii Cortex)	2	2.47	39.49
Wolfiporia cocos (Poria Cocos(Schw.) Wolf.)	2	2.47	41.96
Scutellaria baicalensis (Scutellaria baicalensis Georgi)	2	2.47	44.43
nagaimo (Dioscorea opposita)	2	2.47	46.90
Cornus officinalis Sieb. et Zucc (Cornus officinalis)	2	2.47	49.37
Atractylodes Lancea (Thunb.)DC.(Rhizoma Atractylodis)	2	2.47	51.84
Prunus mume (Dark Plum Fruit)	2	2.47	54.31
Rhizoma Anemarrhenae (Anemarrhena asphodeloides)	2	2.47	56.78

**Table 3:** Herbal medicines in the included studies.

First author year	Name of Herbs	Formulation	Compositions (Latin)	Usage
Xu 2014	Gegen Qin-lian tang	decoction	Radix Puerariae, Picrorhizae Rhizoma, Scutellariae Radix, licorice	200ml, Bid

Tong 2009	Tang Ming-ling pills	pills	Radix Bupleuri, Scutellariae Radix, Radix Rhei Et Rhizome, Aurantii Fructus Immaturus, Picrorhizae Rhizoma, Arum Ternatum Thunb.	6g, Tid

Wangshu 2014	Tang Ke Soft Capsules	capsules	Schisandrae Sphenantherae Fructus	1 capsule, Bid

Zhang 2008	Berberine tablets	tablets	Berberin	0.5g, Bid

Tong 2013	Tang Ming-ling pills(TM81)	pills	Radix Bupleuri, Scutellariae Radix, Radix Rhei Et Rhizome, Aurantii Fructus Immaturus, Picrorhizae Rhizoma, Arum Ternatum Thunb.	6g, Tid

Lian 2011	Tian Qi Jiang Tang Capsules	Capsules	Hedysarum Multijugum Maxim., Trichosanthis Radix, Fructus Ligustri Lucidi, Dendrobium officinale Kimura et Migo, Panax Ginseng C. A. Mey., Lycii Cortex, Picrorhizae Rhizoma, Cornus Officinalis Sieb. Et Zucc., Ecliptae Herba, Galla Chinensis	5 Capsules, Tid

Wang 2014	Six-ingredient rehmannia pill and Folium ginkgo tablet	tablets	Rehmanniae Radix Praeparata, Cornus Officinalis Sieb. Et Zucc.,Cortex Moutan, Rhizoma Dioscoreae, Poria Cocos(Schw.) Wolf., Alisma Orientale (Sam.) Juz.,Ginkgo Folium	Six-ingredient rehmannia pill, 8 pills, Tid Folium ginkgo tablet, 2 tablets, Tid

Lian 2015	Jin li da granules	granules	Panax Ginseng C. A. Mey., Polygonati Rhizoma, Picrorhizae Rhizoma, Sophorae Flavescentis Radix, Ophiopogon japonicus (Linn. f.) Ker-Gawl, Rehmanniae Radix Praeparata, Fallopia multiflora (Thunb.) Harald, Cornus Officinalis Sieb. Et Zucc., Poria Cocos(Schw.) Wol, Eupatorium Fortunei Turcz, Picrorhizae Rhizoma, Anemarrhenae Rhizoma, Epimrdii Herba、Radix Salviae, Radix Puerariae, Litchi Semen, Lycii Cortex	9g/bag, Tid

Ji2013a,b	Xiao Ke pills	pills	Radix Puerariae, Rehmanniae Radix Praeparata, Hedysarum Multijugum Maxim., Trichosanthis Radix, Maydis Stigma, Schisandrae Sphenantherae Fructus, Rhizoma Dioscoreae	5 pills

Hu 2016	Jian Yu Tang Kang granule	tablets	Hedysarum Multijugum Maxim, Rehmanniae Radix Praeparata, Atractylodes Lancea (Thunb.)Dc., Figwort Root, Picrorhizae Rhizoma, Euonymi Alati Ramulus	9 tablets, Tid

**Table 4:** Incidence of adverse events.

	Total events/total number		Risk ratio (95% CI)
	Treatment	Control	
Gastrointestinal reactions	6/1109	9/895	0.67(0.25,1.76)
Rash	1/1109	3/895	0.33(0.44,3.11)
Weakness	2/1109	2/895	1.00(0.15,6.87)
Weight loss	0/1109	1/895	0.33(0.01,8.02)
Frequently urination	1/1109	0/895	3.00(0.12,72.20)
Tinnitus	0/1109	2/895	0.20(0.01,4.08)
Genital swelling	0/1109	1/895	0.33(0.11,8.02)
Elevated blood white blood cell	0/1109	1/895	0.33(0.11,8.02)
Decreased hemoglobin	2/614	0/610	5.00(0.25,102.0)
Elevated urine white blood cell	1/614	1/610	1.00(0.06,15.62)
Total events	13	20	
Incidence of any adverse event		Pooled rate ratio: 0.70 (0.37,1.29)	

**Table 5:** Assessment of quality of evidence.

Question: Should Jinlida plus Antidiabetics versus Antidiabetics be used in Antidiabetics?											
Bibliography: Jinlida plus Antidiabetics versus Antidiabetics for T2DM											

Quality assessment					Summary of Findings						

Participants (studies) Follow up	Risk of bias	Inconsistency	Indirectness	Imprecision	Publication bias	Overall quality of evidence	Study event rates (%)		Relative Effect (95% CI)	Anticipated absolute effects	
							With Control	With Jinlida Plus Antidiabetics		Risk With Control	Risk difference with Jinlida plus Antidiabetics (95% CI)

*HbA1C* (CRITICAL OUTCOME: better indicated by higher values)											

1810 (15 studies) 12 weeks	no serious risk of bias^1^	no serious inconsistency	no serious indirectness	no serious imprecision	undetected	⊕⊕⊕⊕ *HIGH*^1^	902	908	-		The mean hba1c in the intervention groups was *0.65 lower *(0.73 to 0.56 lower)

*FBG* (CRITICAL OUTCOME: better indicated by higher values)											

1820 (15 studies) 12 weeks	no serious risk of bias^1^	no serious inconsistency	no serious indirectness	no serious imprecision	undetected	⊕⊕⊕⊕ *HIGH*^1^	907	913	-		The mean fbg in the intervention groups was *0.89 lower* (1.08 to 0.7 lower)

*2hPG* (CRITICAL OUTCOME: better indicated by higher values)											

1820 (15 studies) 12 weeks	no serious risk of bias^1^	no serious inconsistency	no serious indirectness	no serious imprecision	undetected	⊕⊕⊕⊕ *HIGH*^1^	907	913	-		The mean 2hpg in the intervention groups was *1.62 lower* (1.93 to 1.32 lower)

*HOMA-β* (IMPORTANT OUTCOME: better indicated by lower values)											

992 (7 studies) 12 weeks	serious^1^	no serious inconsistency	no serious indirectness	no serious imprecision^1^	reporting bias strongly suspected	⊕⊕⊝⊝ *LOW*^1^ due to risk of bias, publication bias	499	493	-		The mean homa-*β* in the intervention groups was *0.5 lower* (0.62 to 0.37 lower)

Question: Should Jinlida plus Antidiabetics versus Antidiabetics be used in Antidiabetics?											
Bibliography: Jinlida plus Antidiabetics versus Antidiabetics for T2DM											

Quality assessment					Summary of Findings						

Participants (studies) Follow up	Risk of bias	Inconsistency	Indirectness	Imprecision	Publication bias	Overall quality of evidence	Study event rates (%)		Relative Effect (95% CI)	Anticipated absolute effects	
							With Control	With Jinlida Plus Antidiabetics		Risk With Control	Risk difference with Jinlida plus Antidiabetics (95% CI)

*HOMA-IR* (IMPORTANT OUTCOME: better indicated by lower values)											

1084 (8 studies) 12 weeks	serious^1^	no serious inconsistency	no serious indirectness^1^	no serious imprecision^1^	reporting bias strongly suspected	⊕⊕⊝⊝ *LOW*^1^ due to risk of bias, publication bias	539	545	-		The mean homa-ir in the intervention groups was *1.82 lower* (3.1 to 0.54 lower)

*BMI* (IMPORTANT OUTCOME: better indicated by lower values)											

686 (5 studies) 12 weeks	serious^1^	no serious inconsistency	no serious indirectness	no serious imprecision^1^	reporting bias strongly suspected	⊕⊕⊝⊝ *LOW*^1^ due to risk of bias, publication bias	343	343	-		The mean bmi in the intervention groups was *1.07 lower* (2.08 to 0.06 lower)

^1^No explanation was provided.

## Supplementary Materials

* *Supplementary 1 Supplementary File 1: PRISMA 2009 Checklist.

Supplementary 2 Supplementary File 2: Literature retrieval strategy.
