# Supplementary material for: Does Adjuvant Treatment with Chinese Herbal Medicine to Antidiabetic Agents Have Additional Benefits in Patients with Type 2 Diabetes? A System Review and Meta-Analysis of Randomized Controlled Trials
Source: Evid Based Complement Alternat Med. 2019 May 6;2019:1825750. doi: 10.1155/2019/1825750 (PMC6526576; doi:10.1155/2019/1825750)

Literature retrieval

Detailed search strategies will be made. Additional keywords of relevance could have been detected during the search process and we would have modified electronic search strategies to incorporate these terms. We take PUBMED as an example.

| Step | Keyword | | Number |
| --- | --- | --- | --- |
| #1 | | Search ((((((randomized controlled trial[Publication Type] OR randomized[Title/Abstract] OR placebo[Title/Abstract])))))) | 756295 |
| #2 | | Search ((type 2 diabetes mellitus[Title/Abstract]) OR type 2 diabetes[Title/Abstract]) OR 2 diabetes[Title/Abstract] Sort by: [pubsolr12] | 276822 |
| #3 | | Search ((((Chinese herbal medicine) OR herbal medicine) OR (Chinese medicine) OR (Chinese patent medicine) OR (traditional Chinese medicine )))) | 171523 |
| #4=((#1)  AND  (#2)  AND  (#3)) | | Search (((((((Chinese herbal medicine) OR herbal medicine) OR Chinese medicine) OR Chinese patent medicine) OR traditional Chinese medicine)) AND (((type 2 diabetes mellitus[Title/Abstract]) OR type 2 diabetes[Title/Abstract]) OR 2 diabetes[Title/Abstract])) AND (((((((randomized controlled trial[Publication Type] OR randomized[Title/Abstract] OR placebo[Title/Abstract]))))))) | 347 |


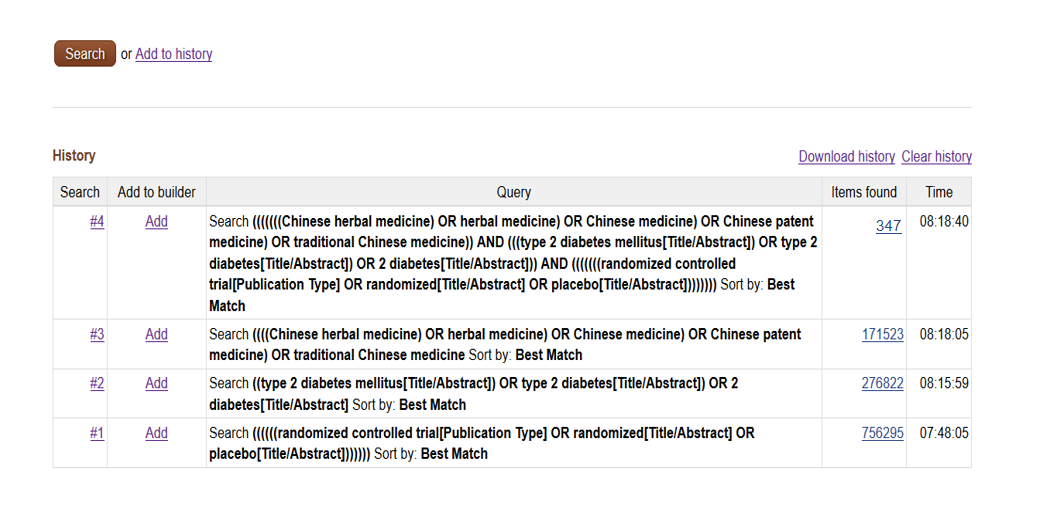

Supplement: Supplementary 2 — Supplementary File 2: Literature retrieval strategy. [file 1825750.f2.doc]
